# Heart rate during work and heart rate variability during the following night: a day-by-day investigation on the physical activity paradox among blue-collar workers

**DOI:** 10.5271/sjweh.3965

**Published:** 2021-06-29

**Authors:** Mette Korshøj, Charlotte Lund Rasmussen, Tatiana de Oliveira Sato, Andreas Holtermann, David Hallman

**Affiliations:** The National Research Centre for the Working Environment, Copenhagen, Denmark; Department of Occupational and Social Medicine, Holbæk Hospital, Hobæk, Denmark; Department of Physical Therapy, Universidade Federal de São Carlos, Brazil; Department of Occupational Health Sciences and Psychology, University of Gävle, Sweden

**Keywords:** aerobic workload, autonomic nervous system, heart rate reserve

## Abstract

**Objectives:**

Contrary to leisure-time physical activity, occupational physical activity (OPA) may have harmful health effects, called the physical activity paradox. A proposed mechanism is that OPA can elevate the heart rate (HR) for several hours per day. We aimed to investigate the association between the mean intensity of OPA and HR variability (HRV) indices the following night.

**Methods:**

Three cohorts (NOMAD, DPhacto, and Physical Workload and Fitness) involving blue-collar workers from different sectors were merged in this study. HR monitors (Actiheart) recorded 24-hour inter-beat intervals (IBI) for up to four consecutive days. The relative intensity of the mean HR during work was estimated by HR reserve (%HRR), and time-domain indices of HRV were analyzed during the following night. Data were analyzed using a multilevel growth model to test the association between mean %HRR during work and HRV indices at night in a day-by-day analysis adjusted for age, BMI, alcohol consumption, smoking, and occupation.

**Results:**

The dataset included a sample of 878 Danish blue-collar workers, with a mean %HRR during work of 31%, and 42% worked at an intensity ≥30%HRR. The multilevel model showed negative within- and between-subject associations between %HRR during work and HRV indices at night.

**Conclusions:**

Our results indicate a higher %HRR during work to associate with lower HRV indices the following night and a higher HR, reflecting an imbalanced autonomic cardiac modulation. This finding supports a high mean HR during work to be a potential underlying mechanism for the harmful health effect of OPA.

High levels of leisure-time physical activity (LTPA) has consistently been associated with better health (1, 2). In contrast, high levels of occupational physical activity (OPA) have recently been linked to increased risk for cardiovascular disease and mortality (3, 4), constituting the physical activity health paradox (5).

The mechanisms behind the potential detrimental health effect of OPA remain to be disentangled, as highlighted in the WHO guidelines for physical activity (6). A conceptual framework providing hypotheses of the characteristics of physical activity and physiological responses that can explain the physical activity health paradox has recently been proposed (5). One of the proposed explanations is that a raised heart rate (HR) during work, for several hours per day, can cause an imbalanced autonomic cardiac activation (7). Imbalanced autonomic cardiac activation is well documented to increase the risk for cardiovascular disease and mortality (8–10). However, this proposed mechanism for the physical activity paradox has not previously been investigated.

The relative intensity of OPA can be assessed by device-worn HR sensors and estimated as percent HR reserve (%HRR), calculated from HR at rest, during working hours, and age-predicted maximum HR (11). The autonomic cardiac activity can also be measured by device-worn HR sensors using HR variability (HRV) analysis. Although some previous studies suggest that higher OPA is related to reduced resting or sleeping HRV (7, 12), studies have not addressed the temporal association between daily changes in the relative intensity of OPA and HRV indices at night. Such analysis should aim to be without confounding from individual factors such as age, sex, cardiorespiratory fitness, and could therefore be performed in an analysis of the day-to-day effect on HRV indices at night from the %HRR during work. No previous studies have investigated the day-to-day effects of the relative intensity of OPA on HRV indices at night using relative aerobic workload (%HRR) as the exposure measure among blue-collar workers, being occupational groups with no or short educations and exposed to manual work.

Thus, this study aimed to investigate the association between the daily mean intensity of OPA and HRV indices the following night among blue-collar workers. We hypothesized a negative association between %HRR and HRV indices the following night.

## Methods

### Study design

This study is based on harmonized data from three occupational field cohorts using continuous sampling of physiological parameters.

### Study population and exclusion criteria

Data from blue-collar workers from the “New method for Objective Measurements of physical Activity in Daily Life” (NOMAD) (13) and “Danish Physical activity cohort with objective measurements” (DPhacto) cohorts (14), and baseline data from a cluster-randomized worksite intervention among cleaners “Physical Workload and Fitness” (15) were merged in this study.

The NOMAD, DPhacto, and Physical Workload and Fitness studies were conducted on workers recruited in Denmark. The inclusion criteria for the three studies were to be (i) allowed to participate during paid working time, (ii) employed for >20 hours per week, and (iii) aged ≥18–≤65 years. Exclusion criteria were: (i) declining to sign the informed consent, (ii) pregnancy, (iii) fever on the testing day, and (iv) an allergy to adhesives. Population and recruitment were described in detail elsewhere (13–15).

Inclusion in the present analysis was based on the following criteria: availability of data on diurnal and nocturnal HR from the 24-hour ECG recordings with a minimum duration of ≥4 hours per day during work time, and a minimum duration of ≥4 hours per night during sleep time.

The Ethics Committee for the Capital Region of Denmark approved the NOMAD, DPhacto, and Physical Workload and Fitness studies (journal number H-2–2011–047, H-2-2012-011 and H-2-2011-116, respectively) and all three studiers were conducted following the Helsinki Declaration. Informed consent was obtained from all participants included in the study.

### Data collection

The technical measurements and questionnaires were similar across studies to enable harmonization of the data. A questionnaire was administered to the workers including age, sex, ethnicity, tobacco use, alcohol consumption, use of anti-hypertensive, anti-depressive, and heart medications. Occupational factors were also collected by the questionnaire and included present occupation (sector), job seniority, and occupational lifting/carrying.

Height (cm) was measured using a scale (Seca, model 123) and weight (kg) was measured by a digital scale (Tanita model BC 418 MA). Body mass index (BMI) was calculated according to the formulae BMI = weight (kg)/height (m^2^). A submaximal fitness test on a cycle ergometer (16) or a submaximal step test (17) was performed to indirectly estimate VO_2_max as a parameter of cardiorespiratory fitness (mlO_2_/min/kg).

A HR monitor (Actiheart system, CamNtech Ltd., Cambridge, UK) was mounted for 24-hour measurements of RR intervals (RRI). The workers were instructed to wear the device continuously for up to four consecutive days, and only to remove it in case of skin irritation. The electrodes were attached to the chest, according to previous recommendations (18). The respiratory rate was not controlled during data collection. Data were sampled at 128 Hz and processed using a band-pass filter (10–35 Hz). The participants also received a diary, in which they were asked to write the time they went out of bed in the morning, started to work, finished work, went to bed in the evening, and periods of non-wear time.

The data were downloaded using the Actiheart software and processed in the Acti4 software (The National Research Centre for the Working Environment, Copenhagen, Denmark and Federal Institute for Occupational Safety and Health, Berlin, Germany) (19). The inter-beat intervals (IBI) were used to calculate the relative aerobic workload during work and leisure and HRV indices.

The relative aerobic workload was estimated by the %HRR, which was calculated using the equation: %HRR=[HR_work_ - (HR_max_ - HR_min_)]/(HR_max_ - HR_min_) × 100 (20). The minimum HR (HR_min_) was defined as the 10^th^ lowest HR for 24 hours including sleep time (21) and the maximal HR (HR_max_) was estimated as HR_max_ = 208 − 0.7 × age (11).

Based on the RRI series, HRV indices were analyzed from 5-minute windows with <5% erroneous RRI, both in the time and frequency domains. Abnormal beats were automatically removed before analyzing parameters of HRV. Besides the time-domain mean inter-beat interval (IBI) (ms), the HRV indices analyzed were the square root of the mean squared differences of successive IBI (RMSSD), and the standard deviation of IBI (SDNN). Mean IBI and SDNN both reflect parasympathetic as well as sympathetic modulation of the cardiac rhythm, but RMSSD is only an indicator of the parasympathetic modulation of cardiac rhythm (22, 23).

### Data analysis

A day-by-day analysis was conducted using a multilevel growth model (ie, linear mixed model), with random intercept and slope, which enabled multiple measurements for each participant. %HRR during work was paired with parameters of HRV during the following night. An unstructured covariance structure was applied. The analysis was performed using unadjusted and adjusted models. In models, the workday was entered as a level-1 predictor. Moreover, weekly mean %HRR during work and the difference between daily and weekly mean %HRR during work were entered as level-2 predictors. This way, weekly mean %HRR during work enabled estimation of the between-subject effect whereas the difference between daily and weekly mean %HRR enabled estimation of the within-subject effect. Moreover, an interaction between weekly mean %HRR during work and workday was entered. In case the interaction term was non-significant (P>0.05), it was excluded from the fully adjusted model. In the adjusted models, age, sex, BMI, alcohol consumption, smoking status, and present occupation (sector) were considered as potential confounders and entered as level-2 predictors. IBI, SDNN, and RMSSD values were considered as outcomes in both crude and adjusted models separately for each outcome. As the SDNN and RMSSD values were not normally distributed, the values were log-transformed. A total of 878 individuals and 1253 observations were included. The statistical models were built with a repeated statement taking the clustering of observations from the same participant into account.

A secondary analysis stratified on mean occupational aerobic workload (</≥30% HRR) across all included workdays was performed to investigate differences in associations between occupational aerobic workload and HRV indices at night. This cut-point of occupational aerobic workload was based on the recommendations from the International Labor Organization (24, 25).

A sensitivity analysis was conducted by stratifying on groups of users versus non-users of anti-hypertensive, anti-depressive, and heart medications. This was done as the effect on HRV may be reversed, concealed, or distorted by the use of these medications (26). Additionally, as the elevation in HR is relative to the individual cardiorespiratory fitness and age, and therefore may identical OPA tasks employ higher strain among less fit/older than more fit/younger workers (27). Thus, interaction terms between mean %HRR during work and cardiorespiratory fitness and age were entered. In the case of a significant interaction term (P<0.05), the fully adjusted model was stratified. By occurrence of extreme values sensitivity analysis excluding those values was performed to investigate whether these results are similar to those not excluding the extreme values.

## Results

**Figure 1:**
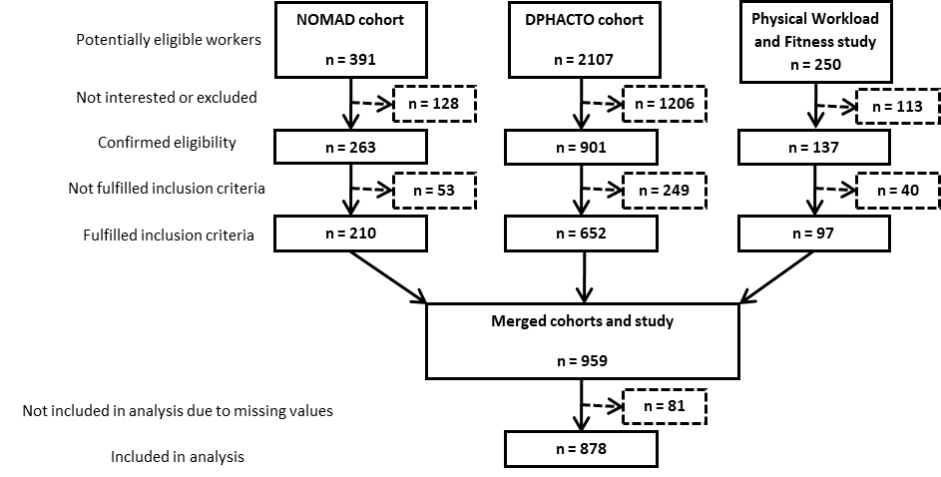
Flowchart of the study population.

From the three cohorts, there were a total of 2748 potentially eligible workers, 1297 of whom were considered eligible; 959 workers fulfilled the inclusion criteria for the current analysis, and 878 workers with complete data were included in the analysis (figure 1).

The included population of 878 participants had a median age of 46.0 years (IQR 39.0–53.0), 46% were female, and the median level of cardiorespiratory fitness was 30.1 mlO_2_/min/kg (IQR 13.6–66.9) (table 1). The median level of self-reported physical demand were 6.0, on a 1–10 scale where 10 was very strenuous, and 77% reported to be exposed to occupational lifting >25% of work time. The median %HRR during work was 31%, indicating moderate to high physical work demands and 52% of the included workers had a mean work HRR ≥30% (24). The majority of the included population were employed in manufacturing, but all of the included participants were classified as blue-collar.

**Table 1:** Baseline characteristics of the included workers (N=878).

Characteristics	N (%)	Median	Q1–Q3
Age (years)		46.00	39.00–53.00
Sex (% female)	403 (45.9)		
Ethnicity			
Danish	731 (85.2)		
Other western countries	44 (5.1)		
No western country	83 (9.7)		
BMI (kg/m^2^)		26.36	23.74–29.86
Smoking (% daily smoking)	246 (28.0)		
Alcohol consumption (drinks in the prior week)			
0	310 (35.3)		
1–5	396 (45.1)		
>5	172 (19.6)		
Cardiorespiratory fitness (mlO_2_/min/kg) ^a^		30.14	25.22–36.74
Using anti-hypertensive, heart or lung medication		78 (9.1)	
Occupational sector			
Cleaning	211 (24.0)		
Manufacturing	490 (55.8)		
Transportation	60 (6.8)		
Health service	16 (1.8)		
Assemblers	29 (3.3)		
Construction	23 (2.6)		
Garbage collectors	24 (2.7)		
Mobile plant operators	6 (0.7)		
Other blue-collar workers	19 (2.2)		
Work hours per week		37.00	37.00–38.00
Job seniority (years)		11.00	5.00–20.00
Self-reported physical work demands (1 none–10 very strenuous)		6.00	5.00–8.00
Occupational lifting (% of work time)			
0–25	184 (22.8)		
>25–50	361 (44.7)		
>50	263 (32.5)		
Relative aerobic workload during work (%HRR)		30.40	25.20–35.52
Mean level of relative aerobic workload (%HRR) ≥30% during work	456 (51.9)		
During night time			
IBI (ms)		984.85	902.68–1076.00
Heart rate (bpm)		60.92	55.76–66.47
SDNN (ms)		61.62	47.71–80.17
Log(SDNN) (ln ms)		4.12	3.87–4.38
RMSSD (ms)		37.32	26.71–53.42
Log(RMSSD) (ln ms)		3.62	3.29–3.98
Measured days (days)		3.00	2.00–4.00

a Indirect estimation of VO_2_max as a parameter of cardiorespiratory fitness (mlO_2_/min/kg). Q1-Q3 first and third quartile

The main effect of the weekday in the crude models was non-significant (IBI -2.14, P=0.24; RMSSD 0.004, P=0.54; SDNN -0.002, P=0.75), indicating no effect of the weekday on the HRV variables. Moreover, the interaction between the weekly mean %HRR at work and weekday was non-significant (IBI P=0.92; RMSSD P=0.97; SDNN P=0.93), indicating that %HRR at work was not associated with the change in nighttime HRV indices over the week. Therefore, these interactions were not included in further analysis.

The multilevel models adjusted for age, BMI, alcohol consumption, smoking status, and the occupational group showed negative associations between weekly mean %HRR during work and HRV indices during the following night, i.e., the between-subject effect (table 2). Workers with higher %HRR at work showed significantly reduced IBI (i.e., elevated HR) and decreased RMSSD. Also, negative associations were seen between %HRR and HRV indices at the worker level, i.e., the within-subject effects (table 2). Specifically, a day with higher %HRR was associated with significantly lower IBI, RMSSD and SDNN the following night.

**Table 2:** Multilevel day-to-day analysis of the association between mean heart rate during work, measured as % heart rate reserve (%HRR), and inter-beat intervals (IBI), and heart rate variability (HRV) indices, during the following night, between- and within-subjects. N=878. [SE=standard error; RMSSD=the log-transformed square root of the mean squared differences of successive IBI; SDNN=the log-transformed standard deviation of IBI].

Mean %HRR during work	Crude model			Adjusted model ^a^		
	
	Estimate	SE	P-value	Estimate	SE	P-value
Between-subject effects ^b^						
IBI (ms)	-6.33	0.62	<0.001	-6.70	0.61	<0.0001
RMSSD (ln ms)	-0.0034	0.002	0.11	-0.005	0.003	<0.05
SDNN (ln ms)	-0.003	0.003	0.20	-0.003	0.002	0.13
Within-subject effects ^c^						
IBI (ms)	-1.64	0.67	0.02	-1.62	0.67	0.02
RMSSD (ln ms)	-0.006	0.002	<0.01	-0.006	0.002	<0.01
SDNN (ln ms)	-0.008	0.003	<0.01	-0.008	0.003	<0.01

a The adjusted model includes adjustment for age, sex, BMI, alcohol consumption, smoking status, and occupational group.

b Weekly mean %HRR at work.

c Difference between weekly mean %HRR and daily mean %HRR of the worker.

Thus, by converting the IBI to HR in beats per minute (bpm), from the between-subject effect, this negative association implies that per one percent higher mean %HRR during work the night-time HR would increase by 0.4 bpm, and 10% higher mean %HRR during work would imply an increase in night-time HR by 4.4 bpm, as the relation between IBI and HR are reciprocal (28).

Stratifying on high (≥30% HRR) and low (<30% HRR) mean %HRR across all measured workdays showed similar associations as for the main analysis (data are now shown). The sensitivity analyses stratified by use of anti-hypertensive medication also showed similar associations’ direction and magnitude as the main analysis.

Moreover, the data included some (16 observations among 4 participants) extreme values of IBI, SDNN and RMSSD which could be considered as outliers, although the corresponding HR estimated from the extreme IBI showed plausible HR values (ranging from 40–93 bpm). Sensitivity analysis excluding the extreme values showed results similar to the reported (table 2), except for the between-subject effects on RMSSD turning non-significant (P=0.07), although the estimate and SE were similar to those reported (table 2).

## Discussion

To investigate both the within- and between-subjects association between the %HRR during work and HRV indices at night among blue-collar workers, we conducted a day-by-day analysis of %HRR during work and HRV indices the following night. Our main findings indicate that a higher %HRR during work is associated with elevated HR (from the lower IBI) and reduced HRV variables the following night. This association was observed both between workers and between days within the worker. This finding suggests that higher %HRR during work is associated with an impaired autonomic cardiac modulation during sleep, and thus indicating a potential underlying mechanism for the hazardous effects of OPA (3, 4), as described in the physical activity paradox (5).

Our main finding of a higher %HRR during work being associated with elevated HR and reduced HRV variables the following night corroborates previous cross-sectional studies indicating that high levels of OPA are associated with autonomic imbalance at rest (29) and during sleep (7). While the previous studies (7) used accelerometer measurements for estimating OPA, we used device-worn HR measurements 24 hours a day, split into work and leisure time to estimate the %HRR during work. The observed reductions in nighttime parameters of HRV (SDNN and RMSSD) indicate a decrease in vagal modulation of the HR (30), being a pre-clinical marker of cardiovascular disease and mortality (8–10). Hence, the autonomic imbalance is a possible pathway for the detrimental effect of OPA on cardiovascular health (7, 29, 31, 32). Thus, the current investigation of the relationship between the %HRR during work and autonomic activity during the following sleep time may help to understand one of the proposed mechanisms in the physical activity paradox, being that high levels of OPA, causing many hours of work per day with elevated HR, which can lead to impaired autonomic cardiac modulation (5).

The explanation for why high levels of OPA can lead to impaired autonomic cardiac modulation is suggested to be the constrained nature of OPA, where the physical activity is determined by the performance of productive work tasks with less individual control over intensity, type and rest breaks (33). Moreover, OPA often occurs over several hours (e.g., 7–8 hours per workday or more) for ≥5 consecutive days per week limiting the possibility for sufficient recovery (33). This is both in line with the allostatic load theory (34), where prolonged exposure to physical stressors with limited recovery could lead to an elevated allostatic load, as reflected in attenuated vagal cardiac modulation. The observation that the workers included in this cohort generally have a low cardiorespiratory fitness will likely further strengthen the association between OPA and impaired autonomic cardiac modulation. This is because workers with low cardiorespiratory fitness will both have an increased relative physiological intensity of OPA (27, 35) as well as a lower capacity to recover from OPA.

Overall, the observed effect sizes from the day-to-day analysis were rather small, which could limit the clinical relevance of our findings. However, the between-subject effects indicated that a 10% increment in weekly mean %HRR was associated with an elevation of the HR during the following night with about 4 bpm. An increase in sleeping HR of 4 bpm is shown to increase the risk of cardiovascular disease (36), and mortality (37, 38).

### Practical implications

The findings of the study support that high mean intensity of OPA is harmfully related to autonomic cardiac modulation the following night. This extends on previous studies finding a similar harmful association when estimating OPA based on accelerometer measurements (7, 39), and studies finding harmful associations between high OPA and cardiovascular disease mortality and all-cause mortality (3, 4). Our study indicates that workplaces ought to make interventions for reducing the mean intensity of OPA among blue-collar workers to prevent negative influences on the cardiovascular system and future health risks. This can be achieved either by (i) reducing the total amount of physical work tasks during the working hours putting a too high load on the cardiovascular system, (ii) implementing sufficient breaks or work tasks which can be performed in a sitting posture, or (iii) workplace initiatives improving the cardiorespiratory fitness of the workers which will reduce the relative cardiovascular load of performing the manual work task and improve the capacity of the workers to recover from the manual work (33).

### Strengths and limitations

The main strength of our study is the day-by-day analysis addressing the temporal association between intensity of OPA and changes in HRV indices the following night, which provides further creditability to the observed association. To our knowledge, no previous study has addressed the effect of daily changes in the intensity of OPA and HRV indices the following night. The homogeneous sample of blue-collar workers minimizes the potential socioeconomic confounding. Furthermore, the 24-hour HR measurements assessments providing both the valid information of the relative physical intensity of OPA and parameters of HRV is an important strength of the study (40). Finally, the sensitivity analysis excluding the extreme values showed results similar to the reported, indicating robust results.

A limitation of the study is the limited measurement duration of one week, which may not be sufficient to capture slow adaptive changes in autonomic activity during sleep related to allostatic load. Furthermore, would future investigations of this association benefit from additional measurement of ambulatory blood pressure to be able to investigate whether increased blood pressures could contribute with a possible explanation of a pathogenic path of high levels of OPA to change HRV parameters. Thus, it is a risk that our study underestimated the observed associations. Moreover, would the estimation of the %HRR benefit from measurement of HR_max_ being more accurate than by general formula estimation. Also, one might speculate that the participating companies providing workers for recruitment in the merged studies, maybe more observant of the work environment, health, and wellbeing than the companies not providing workers for recruitment. This could introduce a selection towards participants being healthier, and working in better environments and thus a risk for our study to underestimate the observed associations. Finally, this study does not hold prospective information and thus causal conclusions cannot be drawn.

### Concluding remarks

The main finding of our study was that higher mean intensity of OPA is associated with elevated HR and reduced HRV indices the following night among blue-collar workers. This association was observed both between workers and between days within the worker. This finding suggests that higher mean intensity of OPA is associated with an impaired autonomic cardiac modulation during sleep. This supports that the high mean intensity of OPA can be an underlying mechanism for the physical activity paradox.
